# 基于探究式教学与批判性思维的仪器分析课程教学实践：以药学专业学生能力培养为例

**DOI:** 10.3724/SP.J.1123.2025.09002

**Published:** 2026-09-08

**Authors:** Bo DING

**Affiliations:** 广州医科大学药学院，广东省分子靶标与临床药理学重点实验室，广东 广州 511436; Guangdong Provincial Key Laboratory of Molecular Target & Clinical Pharmacology，School of Pharmaceutical Sciences，Guangzhou Medical University，Guangzhou 511436，China

**Keywords:** 探究式教学, 批判性思维, 仪器分析, 药学教育, 教学改革, inquiry-based learning, critical thinking, instrumental analysis, pharmaceutical education, teaching reform

## Abstract

仪器分析是药学专业的重要基础课程，其教学效果直接关系到学生的分析检测能力、科研素养与创新思维的培养。当前教学中普遍存在内容体系复杂、原理理解困难以及理论与实验脱节等问题，导致学生缺乏主动探究意识与批判性思维。针对上述问题，本研究以广州医科大学药学院2019‒2023级药学专业学生为对象，探索基于探究式教学与批判性思维培养相结合的教学模式。通过构建“模仿式-比较式-案例式”的递进探究式教学路径，设计问题导向的学习任务与反思性评价机制，旨在引导学生在真实问题情境中实现知识建构与思维提升。教学实践结果表明，该模式有效促进了学生由被动接受向主动探究的转变，显著提升了其分析检测能力、科研素养与创新能力。但仍有部分学生在知识迁移与分析策略选择中表现出惯性思维，批判性推理的深度仍需加强。本研究为仪器分析课程的教学改革提供了系统化路径与实证依据，对培养具有创新能力的高素质药学人才具有重要意义。

## Abstract (en)

Instrumental analysis is a crucial foundational course for pharmacy majors， and its teaching effectiveness directly relates to the cultivation of students’ analytical testing abilities， scientific research literacy， and innovative thinking. Current teaching commonly faces problems such as complex content systems， difficulty in understanding principles， and disconnection between theory and experiments， resulting in students’ lack of active inquiry awareness and critical thinking. To address these issues， this study takes pharmacy students from 2019-2023 classes at the School of Pharmacy， Guangzhou Medical University as subjects， exploring a teaching model that combines inquiry-based teaching with the cultivation of critical thinking. By constructing a stepwise inquiry approach that progresses from imitative to comparative to case-based learning and designing problem-oriented learning tasks and reflective assessment mechanisms， the study aims to guide students in achieving knowledge construction and cognitive enhancement within authentic problem contexts. Teaching practice results indicate that this model effectively promoted students’ transition from passive acceptance to active inquiry， significantly improving their analytical testing abilities， scientific research literacy， and innovative capabilities. However， some students still exhibited habitual thinking in knowledge transfer and analysis strategy selection， and the depth of critical reasoning needs to be further strengthened. This study provides a systematic pathway and empirical evidence for the teaching reform of instrumental analysis courses， and is of significant importance for cultivating high-quality pharmacy professionals with innovative capabilities.

仪器分析是药学专业的重要基础课程，在培养学生的分析检测能力、科学研究素养和实践创新能力方面发挥着关键作用，其教学质量直接影响药学人才培养的整体水平。然而，学生普遍反映该课程学习难度较大。为明确学生在学习过程中的实际困难，本研究以广州医科大学药学院2022级和2023级药学普通班、药学卓越班及临床药学班学生为对象，通过问卷调查收集其在课程学习中遇到的问题。调查结果表明，学生主要面临三方面困难。第一，仪器分析各类方法涉及物理、化学、数学等多学科交叉知识，其基本原理抽象复杂、逻辑关系繁复，学生难以形成系统清晰的知识框架。第二，部分学生在上课时处于“似懂非懂”的状态，实验与理论知识之间的关联理解不足。第三，课程内容涵盖光谱、色谱、电化学、质谱等多种分析技术，知识点数量多、概念相近，学生在学习中易出现混淆。面对上述学习困难，常规以教师讲授为主导的“灌输式”教学模式难以有效解决学生在学习过程中面临的困境。因此，亟须通过创新教学模式与优化教学策略来改进现状，从而切实提升教学效果。

面对上述挑战，传统的以教师讲授为主导的“灌输式”教学模式已难以适应现代药学教育的发展需求。如何在仪器分析课程教学中有效培养学生的批判性思维能力，提升其分析问题、解决问题的综合素养，成为当前教学改革的核心议题。

探究式教学方法起源于20世纪美国教育学界，是一种以学生为主体、注重培养学生主动探究能力的创新教学模式^［1，2］^。该方法强调在教师引导下，学生通过问题探索主动建构知识，教师则主要承担教学设计与学习引导者的角色。在这种教学模式中，学生由被动“灌输式”接受知识转变为主动学习者，以探究性思维方式进行思考和学习。通过将学习过程转化为持续的探究实践，学生在解决真实问题的过程中实现深层次理解，并促进综合能力的全面发展^［3-6］^。

批判性思维是一种兼具合理性、批判性与反思性的高阶思维方式，强调通过深入分析、客观评估和逻辑推理，形成独立、理性的判断与观点^［7］^。批判性思维与创造性思维相辅相成，创造性思维负责产生新颖观点和解决方案，批判性思维则对其进行科学甄别与系统优化，两者协同作用，共同拓展思维的深度和广度，在创新人才培养中发挥重要作用^［8-10］^。从本质而言，批判性思维是智力活动与情感体验的有机统一，是理性反思与价值判断的综合表现。其核心特征包括系统性、延迟性、进阶性、边界性和情境性^［11，12］^。在高等教育实践中，通过完善通识教育体系、倡导慢思考理念、实施阶段递进培养、强化道德规范约束以及创设多元化教学情境等途径，系统培养学生的批判性思维能力，从而使其具备创新型人才培养的核心素养。

基于上述理论基础和现实需求，本研究以药学专业仪器分析课程为实践载体，构建探究式教学与批判性思维培养相结合的创新教学模式。通过理论探索与实践验证相结合的研究路径，旨在为提升仪器分析课程教学质量、培养具备批判性思维和创新能力的高素质药学人才提供科学的理论依据和可操作的实践方案，进而为相关专业课程教学改革提供有益借鉴和参考。

## 1 教学改革综合设计思路

### 1.1 教学目标重构

当前大学生在专业课程学习中过度关注考试分数，对专业知识的掌握呈现碎片化和机械记忆化特征，难以实现理论知识与实践能力的有机融合。传统仪器分析课程教学目标存在明显局限性：单纯以考核分数评价学习成效，忽视了学生发现问题和解决实际问题能力的培养。

因此，需要重新构建以能力导向为核心的教学目标体系。重构后的教学目标应注重培养学生运用仪器分析理论解决实际问题的综合能力。例如，面对校园内桂花飘香的现象，学生应具备独立思考能力，能够选用适宜的仪器分析方法，对香气成分的化学本质进行鉴定；又如，在日常学习和生活中遇到分析检测问题时，能够灵活运用所学知识制定科学的解决方案。这种以问题解决能力为导向的教学目标重构，不仅能够有效对接药学专业人才培养要求和行业实际需求，更体现了现代高等教育中能力培养优于知识传授的教学理念，为培养具有创新思维和实践能力的高素质药学人才奠定基础。

### 1.2 探究式教学模式构建

为了培养药学专业学生发现问题、分析问题和解决问题的能力，本研究在仪器分析教学中构建了以“模仿式-比较式-案例式”为递进层次的探究式教学模式，旨在引导学生主动探究仪器分析相关知识，提升其实践创新能力。

#### 1.2.1 模仿式教学环节

模仿作为人类认知发展的基础方式，在专业技能学习中具有重要作用。在仪器分析实验教学中实施模仿式教学，能够有效帮助学生建立正确的操作规范和思维模式。以HPLC实验为例，教师首先示范“HPLC测定槲皮素含量”的完整操作流程，详细讲解关键技术要点和注意事项。随后，引导学生模仿完成“HPLC测定饮品中咖啡因含量”实验，使学生在模仿过程中逐步理解HPLC的基本原理，掌握标准操作程序，并初步形成独立分析问题的能力。这种由示范到模仿的教学方式，有助于学生快速建立对仪器分析方法的整体认知框架。

#### 1.2.2 比较式教学环节

仪器分析课程涉及光谱分析、电化学分析、色谱分析、质谱分析等多个方面，知识点复杂且相互关联，学生容易产生概念混淆。采用比较式教学方法，通过对比分析相似概念和方法的异同，能够帮助学生准确理解和掌握核心知识点。以紫外-可见光谱分析为例，针对学生普遍困惑的“双光束分光光度法”与“双波长分光光度法”概念，教师通过构建对比表格、绘制原理示意图等方式，从光路设计、检测原理、应用特点等多个维度进行系统比较，引导学生深入探究两种方法的本质差异，从而加深了学生对紫外-可见光谱分析原理和仪器构造的理解，并逐步培养其探究思维能力。

#### 1.2.3 案例式教学环节

仪器分析理论具有高度抽象性，单纯的理论讲授往往难以激发学生的学习兴趣。通过引入真实的分析案例，将抽象理论与具体应用相结合，能够显著地激发学生学习兴趣，从而提升课程教学效果。以“中药材山苍子挥发性成分的GC-MS分析”为例，教师首先引导学生分析山苍子挥发性成分的理化性质，然后指导学生思考选择适宜的分离方法和检测手段，最终确定GC-MS方法进行挥发性成分的分离、检测与鉴定。在此案例分析过程中，学生逐渐学习、理解和掌握气相色谱分离原理、应用范围，以及质谱检测机理与化合物结构鉴定方法，并将其综合应用于山苍子挥发性成分的分析之中。由此可见，案例式教学不仅使学生深入理解色谱-质谱联用技术等仪器分析方法的工作原理和应用价值，更能通过解决实际问题，促进学生主动学习和探究仪器分析课程知识。

### 1.3 批判性思维培养策略

#### 1.3.1 问题导向教学设计

针对药学专业学生初次接触仪器分析课程的认知特点，本研究构建了以问题为导向的教学设计模式。该模式以紫外光谱分析、荧光光谱分析、气相色谱分析、液相色谱分析和电位分析等核心分析技术为载体，通过综合性问题情境，加深学生对基础理论知识的理解与掌握程度，并提升学生在实际问题中的应用能力。例如，“中药材中黄曲霉毒素的含量测定”问题，黄曲霉毒素检测涉及紫外、荧光、液相色谱、质谱等多种分析技术，通过围绕该问题设计教学活动，引导学生从单一分析技术的认知逐步拓展到多种分析技术联用的思考与探究，加深对各类仪器分析方法原理、特点及相互关系的理解，从而实现由单项技术学习向综合分析能力的转化。

#### 1.3.2 科学逻辑思维训练

基于问题导向的教学设计中，培养学生系统分析与解决复杂问题的能力，是促进学生批判性思维形成的关键环节。调研发现，学生在学习仪器分析课程时普遍存在知识点碎片化、缺乏整体分析框架等问题，难以形成系统化的思维路径。为此，本研究构建了科学逻辑思维训练体系，引导学生依据规范的科学分析流程开展问题探究。

以“中药材中黄曲霉毒素含量测定”为例，通过以下逻辑步骤训练学生的系统分析能力。首先，从有机化学角度分析黄曲霉毒素的分子结构特征；其次，探讨其分子结构与光谱响应之间的内在联系；再次，针对中药材复杂的基质特征，分离与纯化过程不可或缺；最后，在上述分析基础上，合理选择HPLC-UV、HPLC-FLD或LC-MS等分析方法，构建完整且可行的黄曲霉毒素含量检测方法。该系统化的思维训练模式不仅有助于学生摆脱碎片化的知识记忆倾向，而且能够促进其形成结构化的问题分析能力，为批判性思维的培养奠定坚实基础。

#### 1.3.3 反思性学习促进机制

在问题导向教学和逻辑思维训练的基础上，建立反思性学习促进机制是提升学生批判性思维能力的重要保障。该机制强调学习者对自身学习过程的持续监控与评价，通过主动反思发现学习中的问题与不足，并及时调整学习策略。具体而言，反思性学习促进机制包括以下3个方面：一是元认知监控，引导学生关注自身的学习状态、思维过程与理解程度；二是问题识别与分析，鼓励学生主动发现学习中的困惑点和知识盲区；三是策略调整与优化，指导学生依据反思结果改进学习方法与推理路径^［10，13］^。

在实际教学中，可通过设置开放性问题促进学生的反思性思考。例如，在掌握多种黄曲霉毒素检测方法后，引导学生思考“哪种分析方法最为恰当、合理”，并进一步拓展至“中药材中人参皂苷的含量检测应采用何种分析技术”等问题。通过这些具有挑战性的案例分析，促使学生反思自己的判断依据和推理逻辑，识别思维过程中的漏洞和认知偏差，并制定相应的改进措施。反思性学习机制不仅有助于学生深化对仪器分析理论知识的理解，更能培养其独立思考和批判质疑的学术品格，为其专业发展和终身学习奠定重要基础。

## 2 课程评价体系改革与实施

### 2.1 形成性评价体系的构建与实施

为科学监测学生在学习过程中的知识掌握与能力发展水平，本研究构建了以探究式教学为核心、以批判性思维培养为导向的形成性评价体系。该体系以“过程监测-阶段反馈-改进提升”为主线，通过阶段性评价节点动态反映学生的学习投入度、知识迁移能力与问题解决水平。

形成性评价指标体系分为3类： （1）知识掌握与理解指标，关注学生对基础概念、原理及分析方法的掌握程度；（2）探究式学习能力指标，重点评估学生提出问题、实验设计与解释实验数据的能力；（3）批判性思维指标，包括对证据判断能力、分析方法的比较能力与逻辑推理的严密性。评价方式采用课堂互动、阶段测试及情境化综合设计能力学习等多元化手段相结合，教师依据学生在各环节中的表现给出形成性评价成绩。

在基于探究式教学与批判性思维培养的仪器分析课程改革实践前（主要教学对象为2019级至2021级药学普通班、药学卓越班和临床药学班），形成性评价主要基于“知识掌握与理解”单一维度，评价方式主要为课堂互动和阶段性测试。教学改革实施后（主要教学对象为2022级和2023级药学普通班、药学卓越班和临床药学班），在继续沿用知识掌握与理解指标的基础上，引入探究式学习能力与批判性思维两个维度，使形成性评价体系更加立体化。同时，在原有的课堂互动和阶段性测试的基础上加入情境化综合题作为新的评价手段，如检出限判别、薄层色谱应用及电泳分析解释等，以评估学生将理论知识应用于实际问题情境的能力。通过上述形成性评价体系，教师能够动态掌握学生在学习过程中的知识发展、思维变化与能力提升状况，及时识别学习瓶颈并调整教学策略，实现“教-学-评”一体化的持续改进与优化。

### 2.2 终结性评价结构的优化与效果导向

终结性评价体系在形成性评价基础上进一步聚焦学生学习成效的综合考核，是检验探究式教学实施效果与批判性思维培养成果的关键环节。优化后的期末评价结构由“基础知识考查题+材料分析题+综合设计题”三部分组成，其中材料分析题则侧重考查学生的分析推理与证据判断能力，综合设计题用于评估学生的知识整合与应用能力。

本研究明确了终结性评价的3项核心效果指标：（1）知识迁移与整合能力：学生能否将所学分析原理灵活应用于新情境问题；（2）逻辑推理与证据判断能力：学生是否能基于实验数据或结构特征做出合理推断；（3）创新与批判性思维表现：学生是否能够提出改进方案或对方法选择进行科学论证。

在探究式教学与批判性思维的仪器分析课程教学改革后，自2021级药学普通班、药学卓越班和临床药学班起，期末终结性评价首次引入综合设计题，要求学生依据化合物的结构特征、分析原理及仪器适配性进行方法选择与实验设计，从而全面评估其知识整合能力与批判性思维水平。在此基础上，2022级药学专业各教学班级又进一步加入材料分析题，以重点考查学生对复杂信息的分析判断与逻辑推理能力。

### 2.3 形成性与终结性相结合的二元评价体系效果

通过纵向跟踪对比发现形成性与终结性评价的结合在以下3个方面显著促进了课程教学目标的实现。（1）反馈闭环建立：形成性评价能够帮助教师及时发现学生在探究式学习过程中的思维盲区，而终结性评价则用于检验相应教学干预与改进措施的实际效果，从而构建“评价-反馈-改进”的闭环机制。（2）批判性思维可测化：通过引入情境化材料分析题与综合设计题作为评价载体，将学生的逻辑推理、证据判断及问题诊断等能力转化为可观察、可量化的评价指标。（3）教学质量提升：教师基于形成性与终结性评价数据动态调整教学策略与方式，实现课程内容、教学方法与评价机制之间的协同优化。总体而言，形成性与终结性相结合的二元评价体系在本研究中实现了对探究式教学与批判性思维培养成效的协同验证，为药学专业仪器分析课程的持续改进提供了可复制性的实践范式。

## 3 教学改革效果分析

### 3.1 2019~2023学年仪器分析课程学生成绩变化趋势

仪器分析课程自2021级药学专业各教学班级起实施探究式教学与批判性思维的教学改革，以促进学生知识迁移与批判性思维能力的发展。改革前（2019‒2020级）课程考核仍以传统的知识记忆与理解为认知目标进行教学；改革后（2021级起），课程考核以知识迁移与综合应用为认知目标进行教学，突出学习过程监测与能力导向的评价体系。

从5个学年仪器分析课程成绩变化趋势看出（图1），在探究式教学与批判性思维教学改革实施前，2019级和2020级药学专业各教学班级仪器分析课程考核成绩中，药学卓越班整体表现相对稳定，而药学普通班和临床药学班的成绩则存在一定程度的波动。2021级药学专业各教学班级中引入探究式与批判性思维教学改革后，3类教学班级的课程成绩均出现一定幅度的下降，表明学生在教学改革初期对新型教学方式的适应性不足，学习方式仍以传统讲授式和知识灌输式为主。对此，授课教师在后续教学过程中根据学生的学习特点和接受能力，对探究式教学与批判性思维教学的实施路径呈现方式进行了适度优化。随着教学模式的逐渐完善，2022级和2023级各教学班级的课程成绩呈现回升趋势，表明学生已逐步适应并能够有效融入探究式与批判性思维导向的教学模式。值得注意的是，2022级各教学班级的考核成绩显著高于其他年级。一方面，这与学生对探究式教学模式的逐步适应密切相关；另一方面，2022年广州医科大学正式进入“双一流”建设行列，当年招生生源质量整体提升，学生的基础知识更加扎实，从而在仪器分析课程的学习与掌握中表现出更强的理解能力与适应能力。总之，5个学年药学专业各教学班级成绩的阶段性波动及后续提升，验证了“评价-反馈-改进”闭环教学机制在探究式教学与批判性思维培养中的有效性。

**图1:**
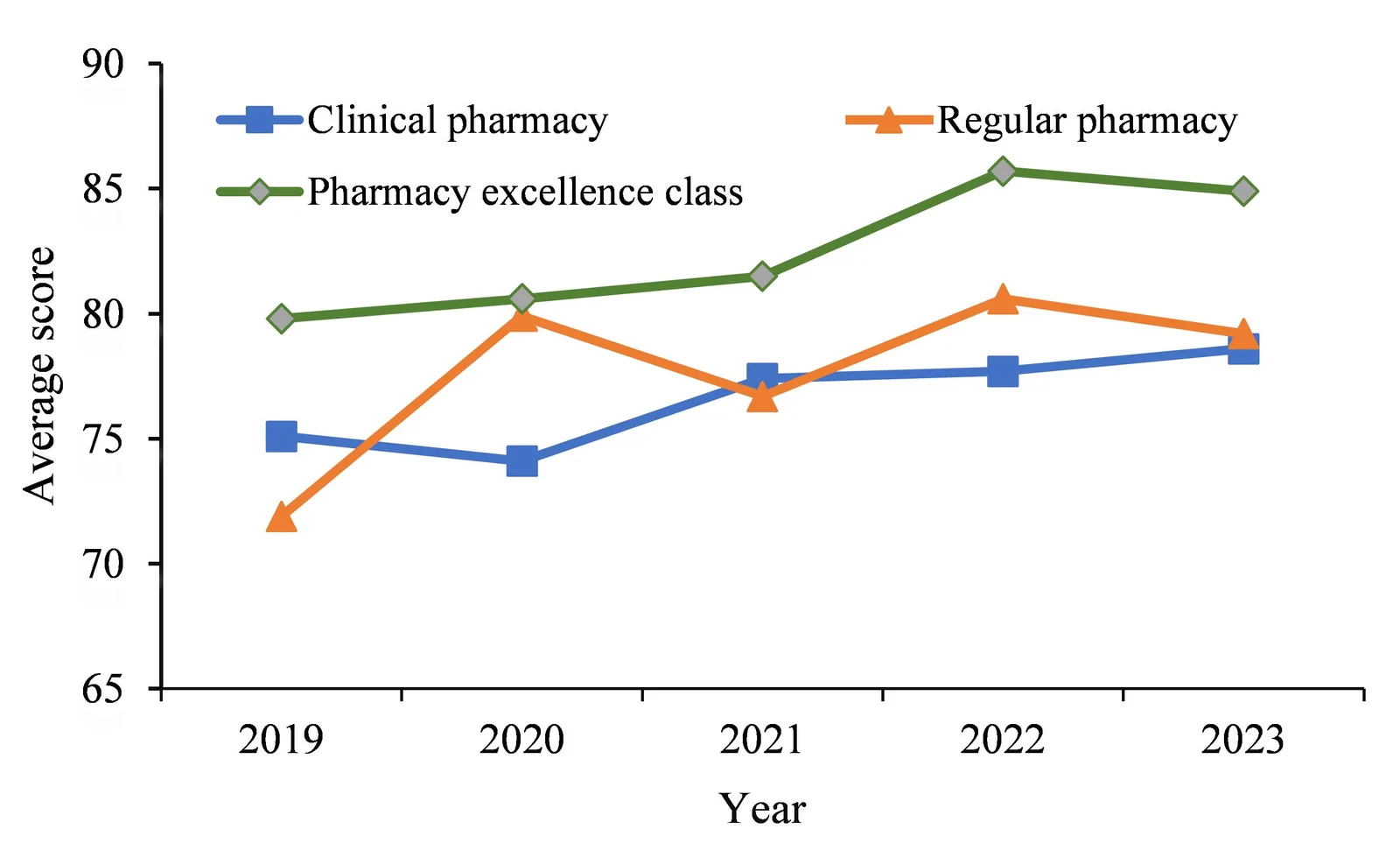
仪器分析课程考核成绩5年变化趋势分析

### 3.2 基于形成性评价的仪器分析课程教学效果分析

为科学评估药学专业各教学班级学生对仪器分析课程相关知识的理解与掌握程度，本研究构建了基于探究式教学理念的形成性评价体系。该体系依托“广州医科大学e学中心”仪器分析线上课程平台，综合课堂活动、任务点学习、课后作业、章节测试、讨论等可量化的指标，从知识掌握、探究式学习能力与批判性思维水平3个维度实施形成性评价。

在此基础上，本研究选取2022级药学专业各教学班级作为研究对象，围绕检出限、薄层色谱（TLC）、电泳分析和原子吸收光谱分析等4个情境应用考核评价指标，考查学生对探究式教学方法的适应程度，并评估学生应用仪器分析理论知识解决实际问题的能力，以判断批判性思维培养的成效。评价结果如表1所示，2022级药学专业各教学班级学生在检出限、电泳分析和原子吸收光谱分析等3个知识点的应用方面表现良好，能够较为有效地实现理论知识与实践问题的结合。然而，在TLC相关题目中，学生的整体正确率明显偏低，表明其对该知识点的理解和掌握仍不充分。进一步对比发现，2022级学生对相对复杂的仪器分析知识点具有较好的理解与应用能力，但对看似基础的TLC相关内容掌握不够扎实。该现象揭示了学生学习过程中存在的一个普遍问题，即对基础性、表面上较为简单的知识点重视不足，进而在综合应用中形成薄弱环节。因此，在后续教学设计与实施中，有必要进一步加强对基础知识的系统梳理与巩固，以提升学生整体的知识结构完整性和应用能力。

**表1:** 2022级药学专业各教学班级情境应用考核评价指标分析

Knowledge point	Accuracy rates/%		
	Clinical pharmacy class	Regular pharmacy class	Pharmacy excellence class
Limit of detection	81.2	85.2	93.3
TLC analysis	34.4	16.0	30.0
Electrophoresis analysis	68.8	85.2	90.0
Atomic absorption analysis	79.7	88.9	90.0

为进一步通过形成性评价分析探究式教学与批判性思维导向的教学改革对仪器分析课程教学成效的影响，本研究又以2023级药学专业各教学班级作为研究对象，同样采用情境应用考核评价指标进行评价。考核内容涵盖光谱分析、电泳分析、毛细管电泳与高效液相色谱（HPLC）对比分析、精确质量测定和离子化技术等5个核心知识点，通过情境应用题评价教学改革的成效（表2）。

**表2:** 2023级药学专业各教学班级情境应用考核评价指标分析

Knowledge points	Accuracy rates/%		
	Clinical pharmacy class	Regular pharmacy class	Pharmacy excellence class
Spectroscopic analysis	7.4	27.1	43.3
Electrophoresis analysis	79.6	79.5	100.0
Comparing CE and HPLC analysis	88.9	77.7	83.3
Accurate mass	9.3	38.8	33.3
Ionization technique	-	64.7	73.3

评价结果显示，学生在不同知识点的掌握程度存在显著差异。在电泳分析、毛细管电泳与HPLC对比分析以及离子化技术等3个知识点方面，2023级学生整体表现较好，能够较为准确地理解相关概念并进行综合应用。然而，在光谱分析和精确质量测定方面，学生的掌握情况明显不足，整体正确率偏低。尤其值得关注的是，2023级临床药学专业学生在这两个知识点上的正确率均低于10%，凸显出光谱分析与质谱分析在仪器分析课程中的难度较高。进一步分析表明，2023级临床药学专业学生在复杂分析技术的理解与应用方面仍存在明显短板，尚未形成灵活运用相关知识解决实际问题的能力。同样，2023级药学普通班和药学卓越班学生在光谱分析和质谱分析的学习过程中，也未能实现知识的系统整合，仍停留在对知识点的碎片化理解阶段，批判性思维能力的培养效果有待进一步提升。

基于上述形成性评价结果，教师将评价数据反馈至教学过程，对学生的薄弱环节进行针对性改进。在后续仪器分析课程教学中，尤其针对光谱分析和质谱分析等高难度内容，优化教学策略，强化理论讲解与实践案例的结合，注重基础知识与高阶分析能力之间的逻辑衔接，促进学生由碎片化认知向系统性理解的转变，从而实现探究式教学与批判性思维培养的动态优化，进一步提升学生分析问题和解决问题的能力。

### 3.3 终结性评价在仪器分析课程教学效果提升中的应用

为系统评估药学专业学生对仪器分析课程核心知识的理解掌握程度及批判性思维能力培养成效，本研究在终结性评价体系中引入材料分析题和综合设计题两大模块，旨在全面考查学生知识掌握的深度与广度，检验探究式教学模式下学生是否达到理论与实践深度融合、知识灵活运用的预期目标。该评价体系对学生的知识迁移能力、证据推理能力及批判性思维水平进行系统测评。

#### 3.3.1 材料分析题教学改革效果分析

材料分析题作为培养学生分析问题和解决实际问题能力的重要评价指标，在不同年级的教学实施中表现出差异化的教学效果。2022级学生的材料分析题聚焦原子吸收光谱法干扰因素识别与处理，该题型设计充分考虑了仪器分析中常见的基体效应、化学干扰和物理干扰等复杂问题。题目满分5分，经统计分析发现，不同班级学生平均得分为2.4~3.1，整体得分率为48%~62%。这一结果表明，2022级学生对原子吸收干扰机制的理论认知尚可，但在实际问题情境中的综合分析和解决能力仍处于中等偏下水平，距离实现仪器分析理论知识与实践应用的有机融合还存在明显差距。

相比之下，2023级学生的材料分析题围绕分析方法评价指标展开，重点考查学生对精密度、准确度、检出限、线性范围等关键参数的理解与应用。同样满分5分的条件下，2023级各班级学生平均得分达到4.5~5分，得分率高达90%~100%，显示出显著优于前一年级的学习效果。这一差异反映出学生对分析方法构建与评价体系掌握程度较高，探究式教学方法在该知识模块的应用取得了较为理想的效果。综合分析两个年级在不同知识点上的表现差异，可以推断探究式教学方法在仪器分析课程改革中已初见成效，但其效果与知识点的复杂程度、抽象程度以及学生的认知基础密切相关。

#### 3.3.2 综合设计题教学改革效果分析

综合设计题作为终结性评价体系的核心组成部分，侧重考查学生对仪器分析课程知识体系的整体把握及多种分析技术的综合应用能力，具有较强的教学效果区分度。基于此，本研究将综合设计题作为主要评价指标，用以衡量探究式教学与批判性思维导向教学改革的实施成效。研究选取连续3个年级的学生作为代表性研究对象进行比较分析：其中，2021级学生以佛手柑内酯和欧前胡素化合物的检测方案设计为评价主题，2022级学生聚焦人参皂苷类化合物的分析方法设计，2023级学生则以芬太尼及其衍生物检测技术为核心内容。通过“练习-考试”对比模式，对学生的知识迁移能力和批判性思维水平进行系统评估。

（1）2021级学生综合设计题表现分析。2021级学生的综合设计题以佛手柑内酯和欧前胡素检测方法设计为核心内容。考前练习阶段，教学团队发布了结构相似的氧化前胡素检测方法设计题，引导学生通过文献调研、方法比较和技术整合等环节，深入理解多种分析技术的联合应用原理。从分子结构特征分析，佛手柑内酯和欧前胡素均属于香豆素类化合物，具有典型的共轭*π*电子体系，在紫外区域呈现特征吸收峰。更重要的是，由于其刚性平面结构特征，在适宜激发条件下能够产生显著的荧光发射现象。基于这一光谱学特性，荧光检测技术凭借其高选择性和高灵敏度优势，理论上最适合复杂基体中此类化合物的识别与定量分析。因此，HPLC-FLD技术应为最优检测方案。

然而，终结评价结果显示，2021级学生在检测方法选择方面仍存在较为明显的认知偏差。如图2所示，3个教学班级中选择理论上最优的HPLC-FLD方法的学生比例均处于较低水平，其中临床药学班为5%，普通药学班为4%，药学卓越班为3%。与此形成对比的是，选择“HPLC”（未明确检测器类型）的学生比例高达65%～72%，表明部分学生在分析方案设计过程中忽视了检测器选择在方法灵敏度与选择性中的关键作用。由2021级学生的终结性评价结果可以看出，学生在分析方案设计这一关键能力环节仍存在明显不足。具体表现如下：学生尚未能基于目标化合物的结构特征与检测需求合理选择最适宜的分析手段，在多种仪器分析技术的综合运用过程中缺乏系统性分析与方法比较意识；同时，面对复杂分析情境，其批判性思维水平仍有待提升，在逻辑推理、证据权衡与技术决策等方面表现出一定局限性。

**图2:**
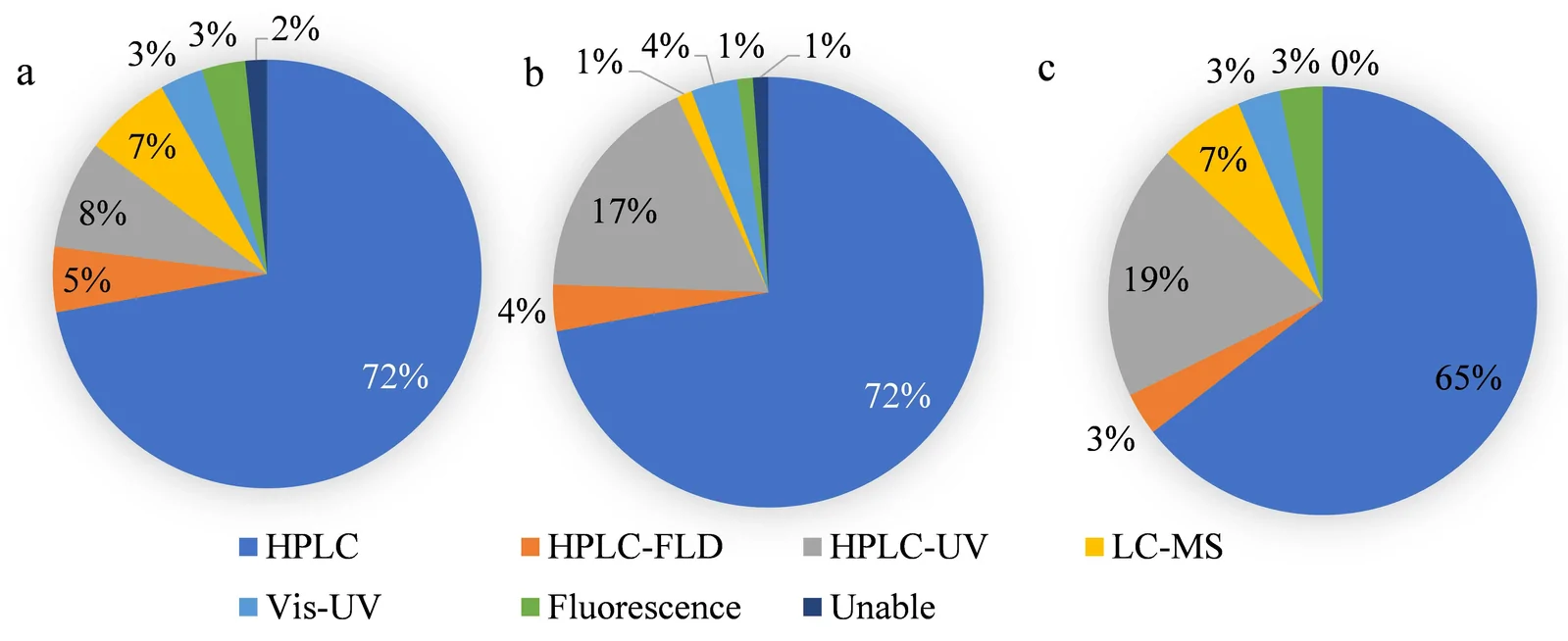
2021级学生在综合设计题中对佛手柑内酯和欧前胡素检测方法的选择分布

（2）2022级学生知识迁移能力测试。为了检验学生能否将“分离-检测”整体分析观念由练习情境有效迁移至新情境，本研究针对2022级学生设计了对比性能力迁移测试。在练习阶段，以佛手柑内酯、氧化前胡素、欧前胡素和异欧前胡素为分析对象。上述化合物均含有共轭发色团，具有明显的紫外吸收特性，从理论上适宜采用HPLC-UV方法进行分析。在期末考试情境中，分析对象更换为人参皂苷类化合物。该类化合物结构中缺乏共轭体系，通常不具备紫外末端吸收特征，因此难以采用常规 UV 检测器进行有效检测。通过这一情境转换，重点考查学生能否基于化合物结构特征差异，合理选择高效液相色谱-蒸发光散射检测器（HPLC-ELSD）或高效液相色谱-示差折光检测器（HPLC-RID）等通用型检测器，从而评价其“分离-检测”整体分析思维的迁移能力。

期末终结评价结果如图3所示，在临床药学班（*n*=58）、普通药学班（*n*=78）和药学卓越班（*n*=30）中，直接作答为“HPLC”的学生比例分别为50.0%、64.1%和33.3%；选择“HPLC-UV”的比例分别为25.9%、21.8%和50.0%。相比之下，能够依据人参皂苷分子结构特征选择HPLC-ELSD、HPLC-RID或LC-MS等更为适宜方法的学生比例明显偏低，3个教学班级中合计仅分别为12.1%、6.4%和16.7%。上述终结性评价结果表明，多数2022级学生在分析方法选择过程中倾向于将“HPLC-UV”作为经验性或习惯性答案进行复现，而未能基于化合物结构特征及其光谱学性质开展证据驱动的逻辑推理。这一现象表明，学生尚未形成“化合物结构特征-检测原理-仪器选择”的完整分析推理链条，在方法决策过程中仍以知识记忆为主，对科学证据的综合分析与论证能力有待进一步加强。

**图3:**
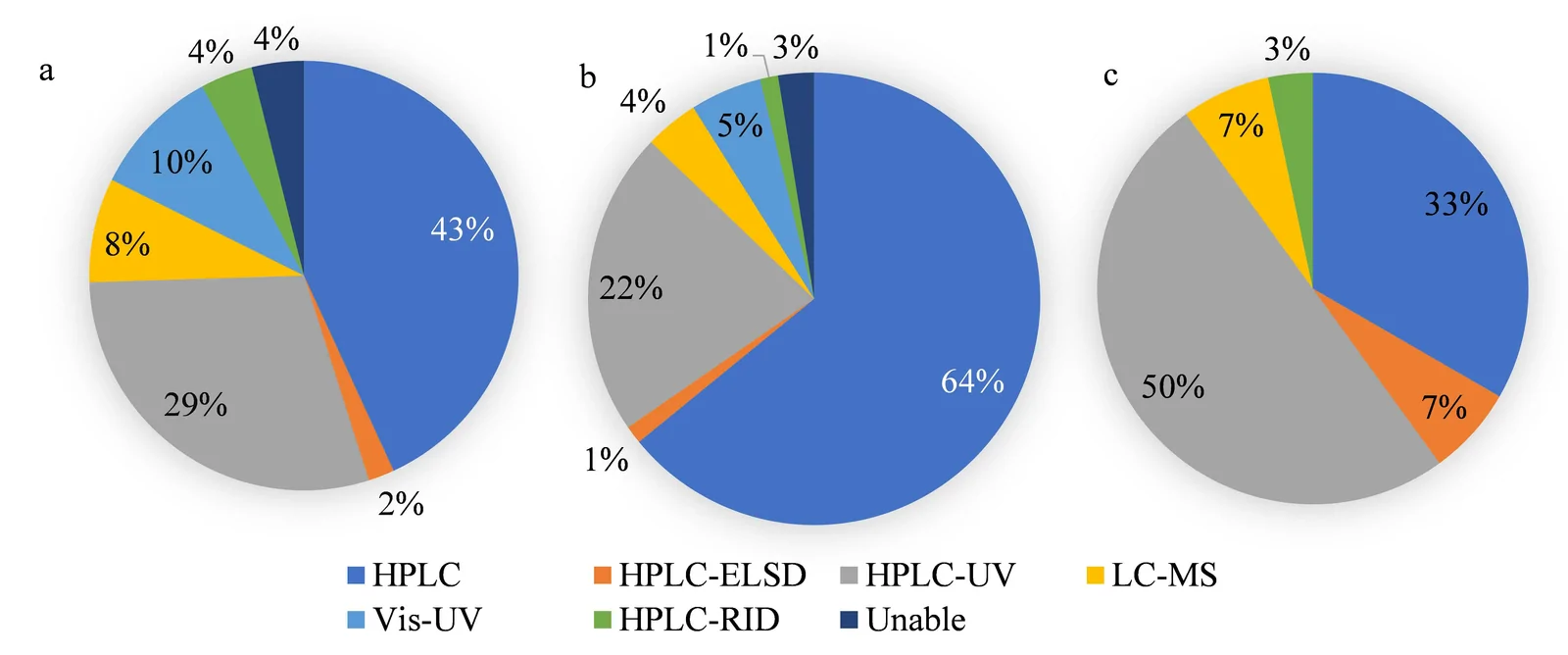
2022级学生在综合设计题中对人参皂苷类化合物检测方法的选择分布

（3）2023级学生持续性问题验证。为进一步验证教学改革成效，2023级学生也接受了类似的能力迁移测试。练习环节以无共轭体系的人参皂苷类化合物为对象，要求设计HPLC-ELSD检测方法；期末考试终结评价中更换为具有共轭发色团的芬太尼类化合物，考查学生能否基于结构特征差异灵活调整检测方案。

终结评价结果显示（图4），2023级学生答题表现与2022级呈现相似问题模式。临床药学班（图4a）中48%学生选择HPLC，33%选择HPLC-ELSD，仅6%选择理论最适宜的HPLC-UV方法。普通药学班（图4b）中40%选择HPLC-ELSD，18%选择HPLC-UV，16%选择LC-MS。药学卓越班（图4c）表现更为不理想，70%学生选择HPLC-ELSD，13%选择LC-MS，14%选择HPLC-UV。

**图4:**
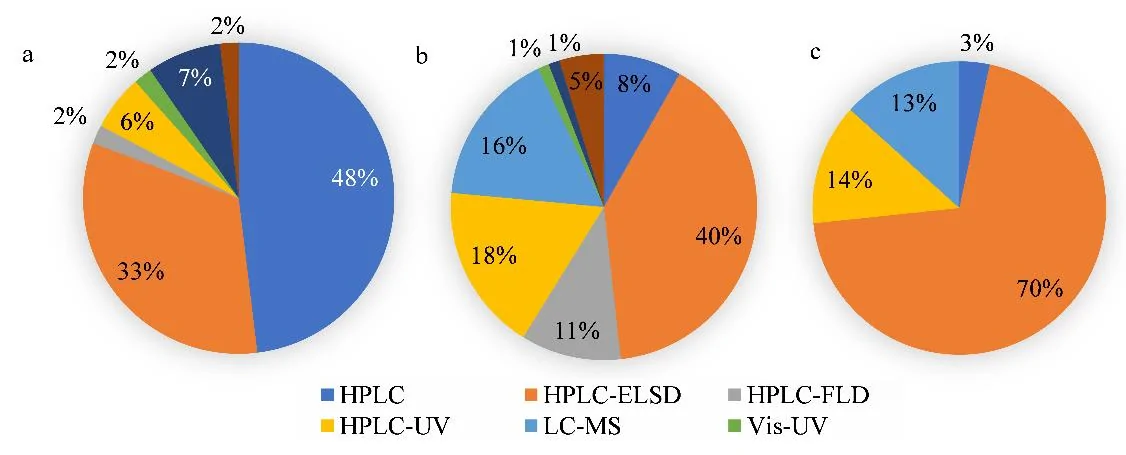
2023级学生在综合设计题中对芬太尼类化合物检测方法的选择分布

3个年级的纵向比较结果显示，33%~70%的学生表现出明显的“惯性思维”特征，倾向于机械地将练习题方案直接移植到考试中，而未能根据目标化合物的结构特点进行针对性分析。这一持续性问题表明，学生普遍缺乏“结构导向的方法学思维”，更多依赖记忆复现而非逻辑推理。尽管历经3年的教学实践调整，学生在批判性思维和知识迁移能力方面仍存在显著不足，未能实现从“知识记忆”向“知识活用”的根本转变。学生缺乏基于化合物分子结构特征进行检测方法合理化选择的科学素养，直接影响了“活学活用、融会贯通”教学目标的达成。

研究发现为后续教学改革提供了重要启示：需要在教学设计中显性化“结构分析-检测原理-方法选择”的逻辑推理过程，通过设置多元化对比案例和结构化评价量表，引导学生建立基于分子结构特征的仪器分析方法选择决策框架，从而切实提升探究式学习能力和批判性思维水平。

## 4 结论与展望

本研究以药学专业《仪器分析》课程为载体，构建了探究式教学与批判性思维培养相结合的教学模式，并通过形成性与终结性评价的有机结合对教学改革效果进行了系统分析。研究结果表明，该教学模式在一定程度上促进了学生由被动接受知识向主动建构知识的转变，显著提升了学生分析问题与解决问题的能力。通过形成性评价的过程监测与即时反馈，以及终结性评价的成果验证，课程改革形成了“评价-反馈-改进”的闭环机制，既保障了教学质量的动态优化，又实现了教学、学习与评价的同频共振。

从具体教学效果看，比较式与案例式探究方法有效缓解了学生在多学科交叉知识学习中的认知障碍，学生逐步形成了“问题识别-假设验证-结果解释”的探究路径。同时，综合设计与材料分析类终结性任务的引入，使批判性思维的评价指标更加显性化与可量化，强化了证据驱动与逻辑推理的能力培养。形成性评价不仅为过程改进提供了数据支撑，也为教师动态调整教学策略提供了科学依据，从而提升了整体教学质量与学习效果。

然而，研究亦发现，学生在知识迁移与方法选择中的“惯性思维”问题仍然存在，批判性思维与证据推理能力尚未完全达到预期目标。这表明教学改革的成效具有一定的阶段性与差异性，仍需进一步深化与完善。未来的教学改革应从以下几个方面推进：（1）在教学设计中强化“结构特征-检测原理-方法选择”的逻辑链条，帮助学生建立基于证据的分析决策框架；（2）针对光谱分析与质谱分析等高难度模块，采用多元化案例与情境化实验设计，推动理论与实践的深度融合；（3）构建纵向持续的能力培养体系，将批判性思维训练贯穿于药学核心课程与科研实践全过程，形成递进式、系统化的培养模式。总体而言，本研究的教学改革实践为药学专业分析科学类课程提供了可复制、可推广的改革路径与实证依据，展现了探究式教学与批判性思维培养在高等药学教育中的融合价值。
